# Interaction between IL-33 Gene Polymorphisms and Current Smoking with Susceptibility to Systemic Lupus Erythematosus

**DOI:** 10.1155/2019/1547578

**Published:** 2019-03-11

**Authors:** Xiaohua Zhu, Lin Xie, Haihong Qin, Jun Liang, Yongsheng Yang, Jinhua Xu, Tiejun Zhang

**Affiliations:** ^1^Department of Dermatology, Huashan Hospital, Fudan University, Shanghai 200040, China; ^2^Department of Epidemiology and Biostatistics, School of Public Health, Fudan University, Shanghai 200040, China

## Abstract

**Aims:**

This study is aimed at exploring the relation between IL-33 single-nucleotide polymorphisms (SNPs) and the risk of systemic lupus erythematosus (SLE).

**Methods:**

SNPStats (online software) was used to test the Hardy-Weinberg equilibrium in controls. Generalized multifactor dimensionality reduction (GMDR) was adopted to screen the preferable interaction between IL-33 SNPs and current smoking.

**Results:**

Logistic regression analysis based on the fundamental data of age, gender, BMI, current smoking, and alcohol drinking showed that both *rs1929992-G* and *rs1891385-C* alleles were correlated with an increasing risk of SLE, the ORs (95% CI) of which were 1.62 (1.21-2.05) and 1.64 (1.22-2.10), respectively. One two-locus model (*rs1929992*×current smoking) had a testing accuracy of 60.11% (*P* = 0.0010). Through an overall multidimensional model, optimum cross-validation consistency was obtained. The analysis indicated that current smoking status influenced the SLE risk depending on the genotypes at *rs1929992*. Pairwise LD analysis indicated that haplotype rs1929992G-rs7044343T was statistically related to the elevating risk of SLE (*P* < 0.05). Those subjects with the G-T haplotype had a higher SLE risk than those with other haplotypes, after correction with factors, including gender, alcohol drinking, age, BMI, and current smoking.

**Conclusions:**

The *rs1929992-G* and *rs1891385-C* allele, interaction between the *rs1929992* gene and current smoking, and haplotype rs1929992G-rs7044343T were all risk factors of SLE.

## 1. Introduction

Systemic lupus erythematosus (SLE) represents a universal type of autoimmune rheumatic diseases. It is featured as the dysfunction of T cell response and B cell activation that leads to the generation of immune complexes in a variety of organs and tissues, which predominantly occurs in young and middle-aged females [1, 2]. In Asians, the SLE incidence was larger than that in European populations, with the gender ratio of 9 : 1 (female : male) [3, 4]. The detailed mechanism of SLE remains elusive. A previous report indicated that genetic predisposition and environmental and hormonal factors participated in the pathogenesis of SLE [5]. Likewise, accumulative evidence highlighted the significance of genetic factors to the susceptibility to SLE [6, 7].

Interleukin-33 (IL-33), as a newly discovered cytokine, is characterized as the member of IL-1 family. It has been reported to exert multiple biological functions, including chronic inflammation and autoimmune disease. The human IL-33 gene is located on chromosome 9 (9p24.1) [8]. Some studies suggested that the serum levels of IL-33 were remarkably upregulated in patients with Behcet's disease (BD), and IL-33 expression in skin tissue was notably higher in BD patients than in the normal individuals [9]. IL-33 was also found to function as the ligand of ST2, and the content in the serum was also associated with disease activity in SLE [10]. Previously, several articles unraveled the correlation between IL-33 gene polymorphisms and autoimmune diseases, such as ankylosing spondylitis, Behçet's disease, and lupus [11–13], and these studies all presented strong susceptibility. But to date, the association between IL-33 polymorphisms and SLE risk remains to be further determined. In addition, the occurrence and development of SLE were affected by not only genetic and environmental factors but also the interaction between the gene and environment. Our study is thus aimed at examining the role of IL-33 SNPs, as well as its interaction with the environment to the susceptibility to SLE in Chinese population.

## 2. Materials and Methods

### 2.1. Subjects

The study sample consists of 846 participants, including 421 patients with SLE and 425 control participants, who were hospitalized in our hospital. All patients diagnosed with SLE were determined by a dermatologist. All SLE patients fulfilled the revised criteria for the diagnosis of SLE by the American College of Rheumatology (ACR) in 1997. Those participants were not included in the control group: (1) suffering from autoimmune diseases or having family history of autoimmune diseases and (2) suffering from critical diseases, including AID, cancer, and some inflammatory diseases. All the subjects enrolled were Han Chinese and they had no genetic relationship with each other. There were no significant differences of age and gender between patients and the control individuals. The index of disease activity was calculated based on the ACR SLE Disease Activity Index (SLEDAI). Informed consent was obtained from each participant.

### 2.2. Genomic DNA Extraction and Genotyping

Blood samples from all participants were acquired and treated with EDTA. Genomic DNA from participants was extracted by using the DNA Blood Mini Kit (Qiagen, Hilden, Germany) according to standard procedures [14] and stored at -20°C for further use. The polymerase chain reaction (PCR) along with restriction fragment length polymorphism (RFLP) was performed for the genotyping of SNPs. The details of nucleotide sequence of primers and SNPs were shown in Table 1. To confirm the genotyping results, sequencing was further conducted on PCR-amplified products.

### 2.3. Statistical Analysis

SPSS 20.0 software (SPSS Inc., IL, USA) was used for statistical analysis. Chi-squared test was analyzed whether the sex differences and the genotype distributions differed from the expected Hardy-Weinberg equilibrium (HWE) and the distribution of genotype and alleles between the SLE and healthy individuals. Data were expressed as mean ± standard deviation. Differences between two groups were compared using *t*-test. Odds ratios (OR) and corresponding 95% CIs were evaluated in the comparison of the genotypes and alleles in the groups of SLE and healthy individuals. The haplotype analysis was performed using PHASE 2.0 (University of Manchester, Manchester, UK). All analyses used the two-tailed estimation of significance. *P* < 0.05 was considered statistically significant.

Generalized multifactor dimensionality reduction (GMDR) was used to analyze the gene-environment interaction; cross-validation consistency, the testing balanced accuracy, and the sign test to assess each selected interaction were calculated. The cross-validation consistency score is a measure of the degree of consistency with which the selected interaction is identified as the best model among all possibilities considered. The testing balanced accuracy is a measure of the degree to which the interaction accurately predicts case-control status with scores between 0.50 (indicating that the model predicts no better than chance) and 1.00 (indicating perfect prediction). Finally, a sign test or a permutation test (providing empirical *P* values) for prediction accuracy can be used to measure the significance of an identified model.

## 3. Results

Distribution of the different demographic characteristics in groups of SLE patients (cases) and normal people (controls) was shown in Table 2. The study population is Han people consisting of 421 SLE patients (137 males and 284 females, 49.8 ± 11.7 years old) and 425 control subjects (135 males and 290 females, 48.6 ± 10.9 years old), and there was *P* > 0.05 in age between the two groups (*P* = 0.199). No significant difference was observed in the parameters including gender, age, BMI, and drinking rates between the two groups (*P* > 0.05). But the current smoking rate was statistically higher in cases than in controls (*P* < 0.05).

In our study, genotype frequencies of these four SNPs in the control group were in line with those expected under the Hardy-Weinberg equilibrium (*P* > 0.05). The frequency for the *rs1929992-G* allele was 30.3% in patients, which was significantly higher than that of 19.8% in controls (*P* < 0.05). The frequency for the *rs1891385-C* allele was 31.1% in cases, which was also significantly higher than that of 20.9% in controls (*P* < 0.05). Logistic regression analysis showed that both the *rs1929992-G* and *rs1891385-C* alleles were related with an enhancing risk of SLE, the ORs (95% CI) of which were 1.62 (1.21-2.05) and 1.64 (1.22-2.10), respectively (Table 3).

High-order interactions were investigated for SLE using the GMDR method. The data in Table 4 showed that one two-locus model (*rs1929992*×current smoking) had a testing accuracy of 60.11% (*P* = 0.001). Moreover, the analysis indicated that current smoking status influenced the SLE risk depending on the genotypes at *rs1929992*.

The result of pairwise LD analysis on four SNPs exhibited that *rs1929992* and rs7044343 within the IL-33 gene were in heavy LD (*D*′ value > 0.75). Haplotype analysis by SHEsis software demonstrated that the haplotype *rs1929992A-*rs7044343C was observed with high frequencies in the two groups, which were 49.47% in the SLE patients and 55.75% in the controls, respectively. The results also indicated that haplotype rs1929992G-rs7044343T was significantly related with the growing risk of SLE (Table 5). Those subjects with the G-T haplotype had a higher SLE risk than those with other haplotypes; after factors including age, BMI, current smoking, gender, and alcohol drinking were excluded.

## 4. Discussion

In our study, we showed that both the *rs1929992-G* and *rs1891385-C* alleles were correlated to the risk of SLE and presented as potential risk factors for developing SLE. However, there was no relationship of rs7044343 and rs10975498 with SLE. Interleukin-33 (IL-33), encoded by the IL-33 gene, functions as a unique cytokine that may induce severe pathological changes and potentially lead to chronic inflammation [15], cancer [16], Behçet's disease [17], and autoimmune disease [18, 19]. Li et al. [19] for the first time found the significance of IL-33 rs7044343 in the development of RA, while the CC genotype of rs7044343 has been linked to the downregulation of serum IL-33 levels. These findings suggested that IL-33 contributed to the pathogenesis of RA. Recently, IL-22 SNPs are reported to be involved in systemic sclerosis and rheumatoid arthritis [20, 21]. Xu et al. [22] in a case-control study revealed that IL-33 rs1929992 was associated with SLE susceptibility according to the result from 371 SLE patients and 408 healthy controls among Chinese Han population. However, they also suggested that, in order to clearly explore the detailed role of this gene on IL-33 expression and the susceptibility to SLE, further functional analysis and the independent case-control study within a large amount of samples from distinct ethnical populations requires to be further conducted, which may eliminate the bias of interaction effect on the current results. In terms of rs1891385, another finding based on 257 SLE patients and 283 healthy controls in the Chinese population showed that rs1891385A/C polymorphism of IL-33 was one of the critical factors affecting the risk of SLE [23]. Importantly, the rs1891385C allele resulted in the higher risk of SLE than rs1891385-A allele did.

Basically, the pathogenesis of SLE is appreciated to be a complicated process, which is associated to environmental and genetic factors [13]. Some studies suggested that smoking may increase the risk of SLE [24, 25], while accumulative evidence shed light on the influence of gene-smoking interaction on SLE [26, 27]. Kiyohara et al. [26] reported that the relation between cigarette smoking and SLE varied due to genetic factors among female Japanese subjects. Our initial evidence demonstrated the impact of IL-33 and current smoking interaction on SLE risk in the Chinese population. The results also indicated that haplotype rs1929992G-rs7044343T may exert as a major determinant to the growing risk of SLE. Those subjects with the G-T haplotype had a higher SLE risk than those with other haplotypes, after correction for BMI, alcohol drinking, current smoking, gender, and age. However, the limitation in our study still existed; extra environmental factors as well as the interactions with SNPs ought to be further identified. In conclusion, our data demonstrated that the rs1929992-G and rs1891385-C alleles, interaction between the rs1929992 gene and current smoking, and haplotype rs1929992G-rs7044343T were all correlated to the rise of SLE risk.

## Figures and Tables

**Table 1:** Description and primer sequences designed for sequencing 4 SNPs.

SNPs	Chromosome	Major/minor alleles	Primer (5′→3′)
**rs1929992**	9:6251588	A/G	Forward: 5′-CTTCTGCCCATTTGCAGCTGAT-3′
			Reverse: 5′-TCTTGAAGTCATSATCAACTTGGAACC-3′
rs7044343	9:6254208	C/T	Forward: 5′-ACGTTGGATGTTGGGTGACACTATGAGTGG-3′
			Reverse: 5′-CAGGAAAGCTGATGCCCCAGTAT-3′
**rs1891385**	9:6219845	A/C	Forward: 5′-CAAACAAGCAACAAAATCCCACTCA-3′
			Reverse: 5′-AATGGGACCTGACCCTGGACTT-3′
rs10975498	9:6226688	C/T	Forward: 5′-CCATCTCTGTTCTAGCATCTCCTTCTACC-3′
			Reverse: 5′-AAGCCACAAAGCCTTTGTGTATTAGAAC-3′

**Table 2:** General characteristics of study participants in the case and control group.

Variables	SLE cases (*n* = 421)	Controls (*n* = 425)	*P* values
Age (years) (means ± SD)	49.8 ± 11.7	48.6 ± 10.9	0.199
Females, *N* (%)	284 (67.5)	290 (68.2)	0.809
Current smoking, *N* (%)	128 (30.4)	80 (18.8)	0.000092
Alcohol drinking, *N* (%)	142 (33.7)	155 (36.5)	0.404
BMI (kg/m^2^) (means ± SD)	23.1 ± 7.4	23.9 ± 7.8	0.126
Duration of disease (years)	5.8 (2.7-8.1)		
SLEDAI	15 (5-29)		

SLEDAI: Systemic Lupus Erythematosus Disease Activity Index.

**Table 3:** Association analysis for 4 target SNPs within the IL-33 gene and SLE risk.

SNPs	Genotypes or alleles	Frequencies *N* (%)		OR (95% CI)^∗^	HWE test for controls
		Controls (*n* = 425)	SLE cases (*n* = 421)		
**rs1929992**	AA genotype	277 (65.2)	209 (49.6)	1.00 (ref)	0.299
	AG genotype	128 (30.1)	169 (40.2)	1.51 (1.17-1.89)	
	GG genotype	20 (4.7)	43 (10.2)	2.04 (1.34-2.78)	
	A allele	682 (80.2)	587 (69.7)	1.00 (ref)	
	G allele	168 (19.8)	255 (30.3)	1.62 (1.21-2.05)	

rs7044343	CC genotype	244 (57.4)	220 (52.2)	1.00 (ref)	0.170
	CT genotype	149 (35.1)	159 (37.8)	1.26 (0.83-1.85)	
	TT genotype	32 (7.5)	42 (10.0)	1.58 (0.76-2.42)	
	C allele	637 (74.9)	599 (71.1)	1.00 (ref)	
	T allele	213 (25.1)	243 (28.9)	1.28 (0.81-2.01)	

**rs1891385**	AA genotype	270 (63.5)	201 (47.7)	1.00 (ref)	0.201
	AC genotype	132 (31.1)	172 (40.9)	1.51 (1.27-1.89)	
	CC genotype	23 (5.4)	48 (11.4)	2.12 (1.43-2.88)	
	A allele	672 (79.1)	580 (68.9)	1.00 (ref)	
	C allele	178 (20.9)	262 (31.1)	1.64 (1.22-2.10)	

rs10975498	CC genotype	249 (58.6)	224 (53.2)	1.00 (ref)	0.593
	CT genotype	150 (35.3)	162 (35.5)	1.25 (0.96-1.63)	
	TT genotype	26 (6.1)	35 (8.3)	1.36 (0.89-1.88)	
	C allele	648 (76.2)	610 (72.4)	1.00 (ref)	
	T allele	202 (23.8)	232 (27.6)	1.29 (0.95-1.69)	

^∗^Adjusted for age, gender, BMI, current smoking, and alcohol drinking.

**Table 4:** GMDR analysis for the best interaction combination models.

Locus no.	Best combination	Cross-validation consistency	Testing accuracy	*P* values ^∗^
Gene-gene interactions^∗^				
2	**rs1929992**×**rs1891385**	8/10	0.5399	0.0547
3	**rs1929992**×**rs1891385**×rs7044343	9/10	0.4958	0.1719
4	**rs1929992**×**rs1891385**×rs7044343×rs10975498	7/10	0.4958	0.3770
Gene-current smoking interactions^∗∗^				
2	**rs1929992**×current smoking	9/10	0.6011	0.0010
3	**rs1929992**×rs7044343×current smoking	7/10	0.5399	0.1719
4	**rs1929992**×rs7044343×**rs1891385**×current smoking	6/10	0.5399	0.3770
5	**rs1929992**×rs7044343×**rs1891385**×rs10975498×current smoking	5/10	0.4958	0.4258

^∗^Adjusted for age, gender, BMI, alcohol drinking, and current smoking. ^∗∗^Adjusted for age, gender, BMI, and alcohol drinking.

**Table 5:** Haplotype analysis on association of the IL-33 gene and SLE risk.

*Haplotypes* (**rs1929992** and rs7044343)	Frequencies		OR (95% CI)	*P* values^∗^
	Case group	Control group		
A-C	0.4947	0.5575	1.00	—
A-T	0.2350	0.2273	1.31 (0.70-1.99)	0.481
G-C	0.1984	0.1807	1.20 (0.65-1.93)	0.532
G-T	0.0719	0.0345	1.65 (1.06-2.21)	<0.001

^∗^Adjusted for gender, age, current smoking, alcohol drinking, and BMI.

## Data Availability

The data used to support the findings of this study are available from the corresponding authors upon request.
